# Supplementary material for: Coordinated spinal locomotor network dynamics emerge from cell-type-specific connectivity patterns
Source: bioRxiv. 2025 Mar 1:2024.12.20.629829. Preprint. [Version 2] doi: 10.1101/2024.12.20.629829 (PMC11888175; doi:10.1101/2024.12.20.629829)
Supplement: Supplement 1 [file NIHPP2024.12.20.629829v2-supplement-1.pdf]

bioRxiv preprint doi: https://doi.org/10.1101/2024.12.20.629829; this version posted March 1, 2025. The copyright holder for this preprint (which was not certified by peer review) is the author/funder, who has granted bioRxiv a license to display the preprint in perpetuity. It is made available under aCC-BY 4.0 International license.

Supplemental Figures

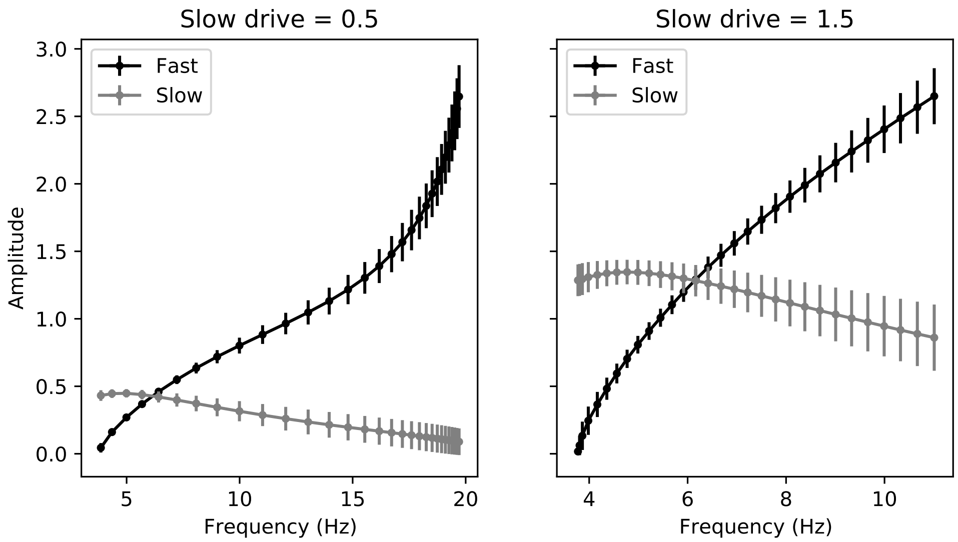

Supplemental Figure S1: Recruitment of fast module at high frequencies inhibits slow module. Data from Figure 3B plotted along paths with constant drive to the slow population (left: small drive; right: large drive) as drive to the fast population is varied.

bioRxiv preprint doi: https://doi.org/10.1101/2024.12.20.629829; this version posted March 1, 2025. The copyright holder for this preprint (which was not certified by peer review) is the author/funder, who has granted bioRxiv a license to display the preprint in perpetuity. It is made available under aCC-BY 4.0 International license.

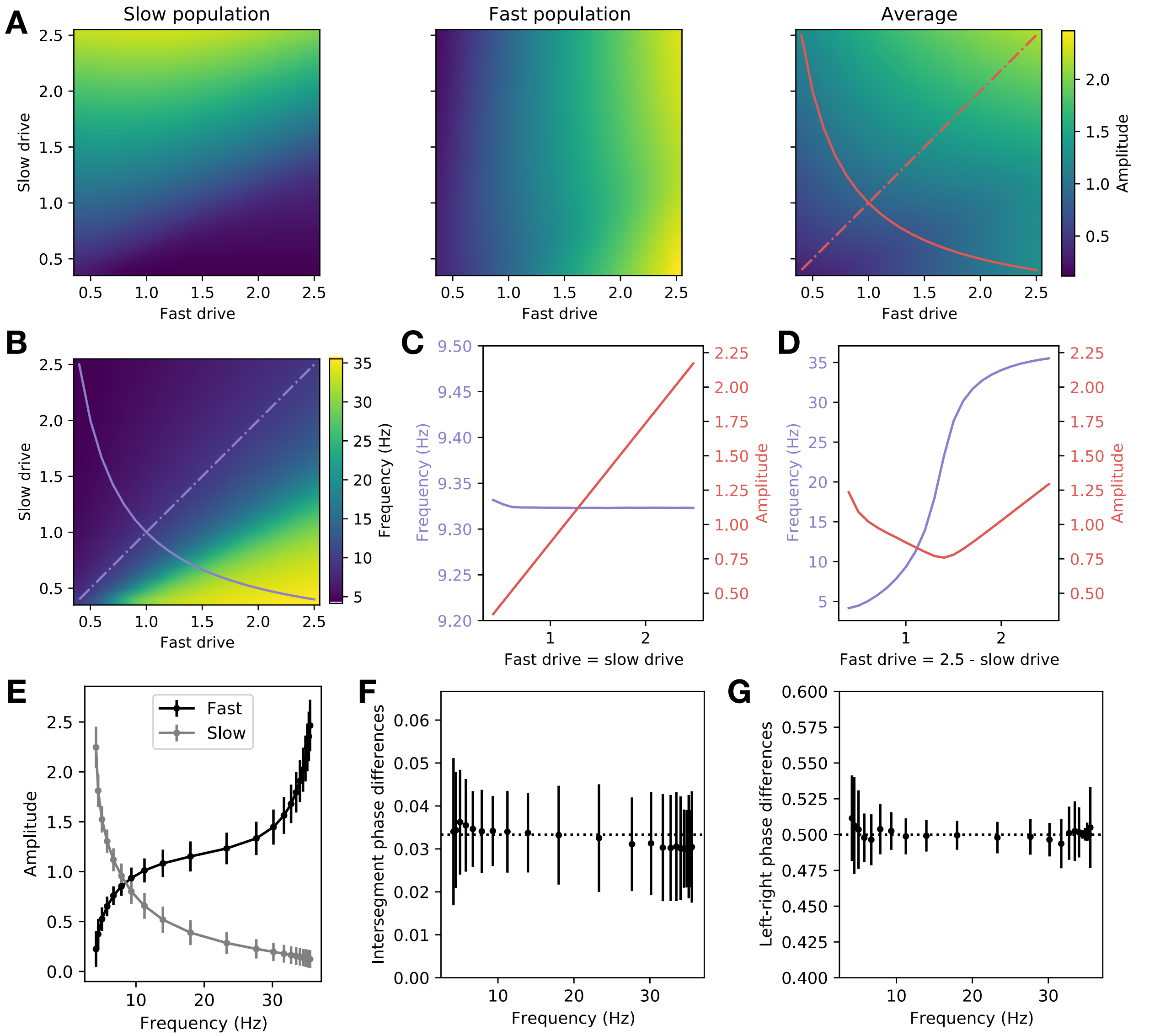

Supplemental Figure S2: Speed-module recruitment enables coordinated locomotion with frequency and amplitude control in an eight-population model **A**: Amplitude of the fast population (left), the slow population (center), and averaged over the fast and slow populations (right). **B**: Levels of tonic drive to the fast and slow populations determine locomotion frequency. **C**: Average amplitude and frequency along the path shown as a solid line in (A) and (B). **D**: Average amplitude and frequency along the path shown as a dash-dot line in (A) and (B). **E**: Frequency-dependent recruitment of fast and slow units as a function of locomotion frequency. **F**: Phase difference between units within each segment (dotted line corresponds to half of a period). **G**: Phase difference between pairs of units on the same side in adjacent segments (dotted line corresponds to $1/N$, where $N = 30$ is the number of segments). Error bars in all panels denote standard deviation across units.

bioRxiv preprint doi: https://doi.org/10.1101/2024.12.20.629829; this version posted March 1, 2025. The copyright holder for this preprint (which was not certified by peer review) is the author/funder, who has granted bioRxiv a license to display the preprint in perpetuity. It is made available under aCC-BY 4.0 International license.

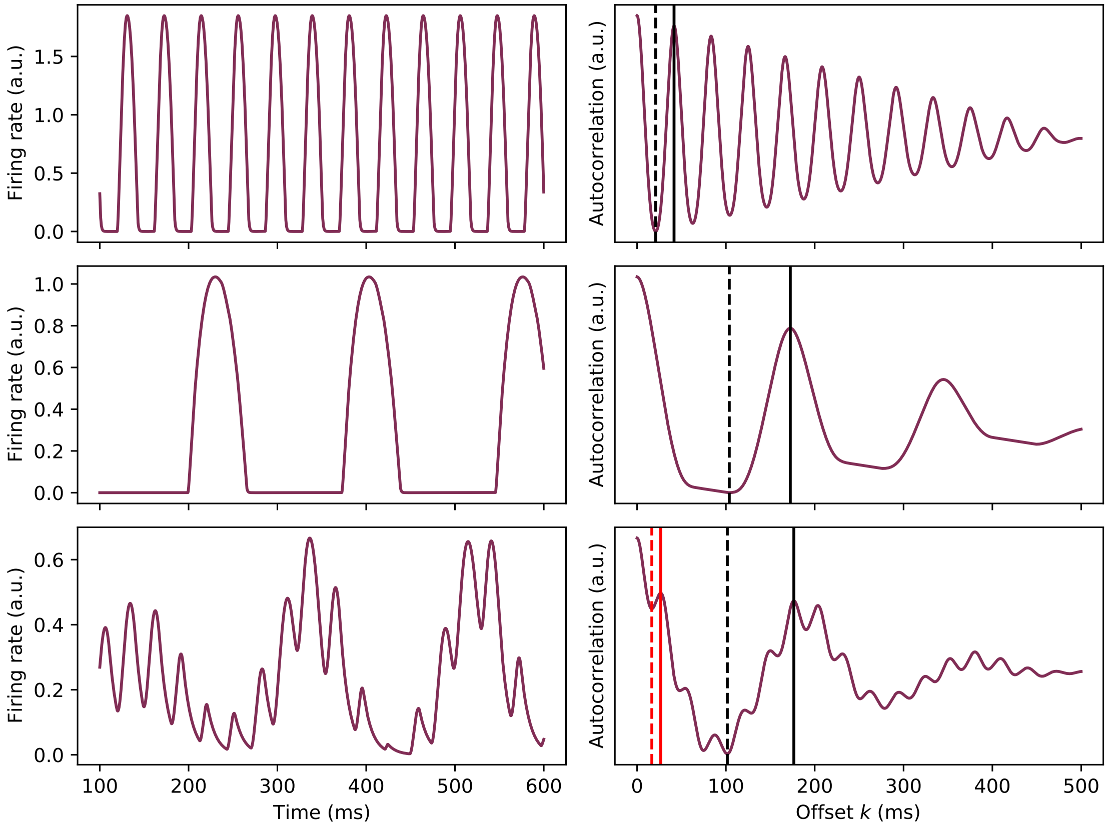

Supplemental Figure S3: Frequency determination from time series is performed through calculating the period from the autocorrelation spectrum. **A**: An example of a high-frequency rate time series from a single unit in the 8-population model. **B**: The autocorrelation spectrum corresponding to the time series in (A). The global minimum and resultant period are marked in dashed and solid lines, respectively. **C**: An example of a low frequency time series from a single unit in the 8-population model. **D**: The autocorrelation spectrum for the time series in (C). The global minimum and resultant period are marked in dashed and solid lines, respectively. **E**: An example time series without a single dominant frequency in the 8-population model. The failure was brought about by increasing the global strength of excitatory connections to 0.5 and the modularity to 0.4. **F**: The autocorrelation spectrum of the the time series in (E). The global minimum and resultant period are marked in dashed and solid black lines, respectively. The first local minimum and resultant period are marked in dashed and solid red lines, respectively.
